# scHaplotyper: haplotype construction and visualization for genetic diagnosis using single cell DNA sequencing data

**DOI:** 10.1186/s12859-020-3381-5

**Published:** 2020-02-01

**Authors:** Zhiqiang Yan, Xiaohui Zhu, Yuqian Wang, Yanli Nie, Shuo Guan, Ying Kuo, Di Chang, Rong Li, Jie Qiao, Liying Yan

**Affiliations:** 1 Center for Reproductive Medicine, Department of Obstetrics and Gynecology, Peking University Third Hospital, Beijing, 100191 China; 2 Key Laboratory of Assisted Reproduction, Ministry of Education, Beijing, 100191 China; 3 Beijing Key Laboratory of Reproductive Endocrinology and Assisted Reproduction, Beijing, 100191 China; 4 Peking-Tsinghua Center for Life Sciences, Peking University, Beijing, 100871 China; 5 Academy for Advanced Interdisciplinary Studies, Peking University, Beijing, 100871 China; 6 Beijing Advanced Innovation Center for Genomics (ICG), Peking University, Beijing, 100871 China

**Keywords:** Single cell DNA sequencing, Haplotyping, Preimplantation genetic diagnosis, Single gene disorder

## Abstract

**Background:**

Haplotyping reveals chromosome blocks inherited from parents to in vitro fertilized (IVF) embryos in preimplantation genetic diagnosis (PGD), enabling the observation of the transmission of disease alleles between generations. However, the methods of haplotyping that are suitable for single cells are limited because a whole genome amplification (WGA) process is performed before sequencing or genotyping in PGD, and true haplotype profiles of embryos need to be constructed based on genotypes that can contain many WGA artifacts.

**Results:**

Here, we offer scHaplotyper as a genetic diagnosis tool that reconstructs and visualizes the haplotype profiles of single cells based on the Hidden Markov Model (HMM). scHaplotyper can trace the origin of each haplotype block in the embryo, enabling the detection of carrier status of disease alleles in each embryo. We applied this method in PGD in two families affected with genetic disorders, and the result was the healthy live births of two children in the two families, demonstrating the clinical application of this method.

**Conclusion:**

Next generation sequencing (NGS) of preimplantation embryos enable genetic screening for families with genetic disorders, avoiding the birth of affected babies. With the validation and successful clinical application, we showed that scHaplotyper is a convenient and accurate method to screen out embryos. More patients with genetic disorder will benefit from the genetic diagnosis of embryos. The source code of scHaplotyper is available at GitHub repository: https://github.com/yzqheart/scHaplotyper.

## Introduction

Single cell DNA sequencing examines genome sequence information obtained from the small amount of starting materials present in a single cell, thus enabling several applications in medical genomics for identifying conditions given the limited amount of available materials; these include the genetic diagnosis of human gametes, zygotes, and blastomeres of embryos [1–4]. Before genetic analyses, a whole genome amplification (WGA) procedure is usually required to yield sufficient DNA for sequencing. Unfortunately, WGA produces several artifacts in amplification cycles, including allelic drop-out (ADO), base replication errors, and non-uniform coverage of the genome [5]. These artifacts will affect the genotyping and haplotyping of single cells. Tracking the transmission of disease allele(s) is imperative in the genetic diagnosis of embryos; however, WGA artifacts may challenge the detection of the disease allele(s).

An alternative method for detecting disease allele transmission is haplotyping by using the disease allele-linked SNPs on one chromosome to infer the transmission of the disease allele. The computational haplotyping methods used for bulk DNA sequencing data have been well established. Pedigree phasing with related individuals is very efficient and accurate in haplotyping research [6]. By leveraging the relations of the individuals and identical-by-descent between individuals within families, the linked SNPs on one chromosome have been identified [7, 8]. O’Connell et al. designed the pipeline suitable for different types of relatedness [9]. Additionally, the haplotype can be constructed even some members are missing in the family [10, 11]. Several applications, such as HaploForge, can be used to construct and visualize the haplotype simultaneously [12]. In population genetics studies with unrelated individuals, the majority of the phasing methods rely on the modelling of haplotype frequencies [13–16], which were well described by Browning et al. [17]. Long read sequencing is a powerful method to determine the linked SNPs within a sequencing fragment. In haplotype assembly by using long sequencing reads, Minimum Error Correction (MEC) is one of the most successful approaches (along with some different types of developed tools) to resolve the haplotype of the individuals [18–23]. However, these methods for bulk DNA sequencing data are not suitable for single cell data because of the error-prone of genotypes caused by the ADO and amplification errors [24]. While there are methods that can construct haplotypes from single cells by leveraging the long amplification fragments produced by multiple displacement amplification (MDA) [24], these methods may not be suitable for other types (such as MALBAC, DOP-PCR) of single cell DNA sequencing data.

Here, we describe a method, scHaplotyper, that constructs haplotype profiles of embryos from single cell DNA sequencing data based on the Hidden Markov Model (HMM). This method is suitable for different types of single cell DNA sequencing data. We applied this method to the clinical genetic diagnosis in two cases of preimplantation embryos derived from patients with monogenic disease and demonstrate the accurate diagnosis of the disease allele carrier status of preimplantation embryos. Our method broadens the application of NGS in precision medicine.

## Implementation

**Fig. 1:**
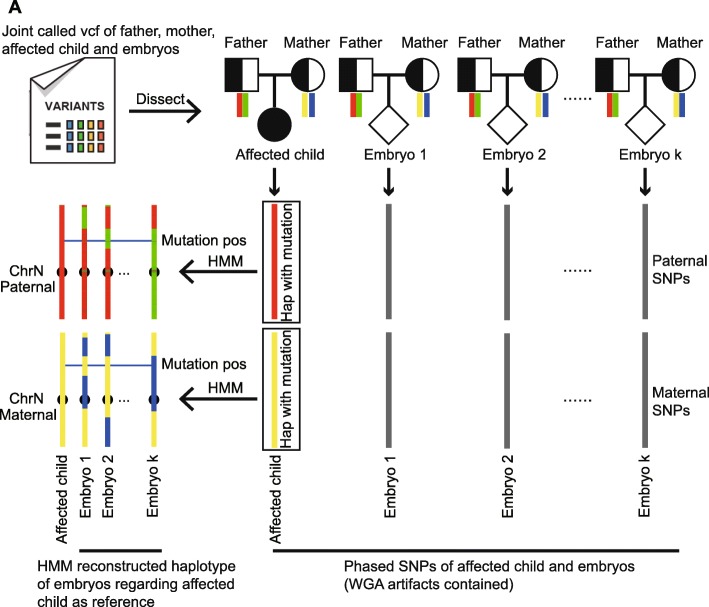
**a** The principle of scHaplotyper. First, trio father-mother-child and father-mother-embryo “core families” are constructed, and the affected child and embryos are phased to paternal and maternal inheritance. The affected child-inherited paternal and maternal haplotypes are shown as red and yellow, respectively. Second, the haplotypes of the embryos are reconstructed by regarding the affected child as a reference. The horizontal line indicates the mutation position on the chromosome. The embryo is diagnosed as carrying paternal and/or maternal mutations when the embryo inherits the same paternal and/or maternal haplotype block as the affected child at the mutation position on the chromosome

In the clinic, sequencing samples used in preimplantation genetic diagnosis (PGD) cases usually include DNA obtained from the trio-family (including the father, mother and affected child/abortus) and amplified products of blastomeres of the embryos derived from the parents. The core functionality of scHaplotyper is designed to infer the haplotype of each embryo to identify the carrier status of the disease allele. The scHaplotyper that infers the carrier status of the disease allele of each embryo includes the following two steps (Fig. 1).

First, father-mother-child and several father-mother-embryo “core families” were constructed, and the child and embryos were then phased according to the Mendelian laws of segregation. Specifically, for each core family, the SNPs in child/embryo were assigned to paternal or maternal origin. For example, if the genotypes of father, mother and child at one SNP loci are “AA”, “AT”, and “AT”, respectively. The “A” in child is assigned to paternal SNP and the “T” is assigned to maternal SNP by applying for the Mendelian laws of segregation. In this regard, the SNPs inherited from the father and mother were identified in the child and embryos. The raw paternal and maternal haplotypes of child and embryos were identified.

Second, among the WGA products of the embryos, which may contain thousands of artifacts, the haplotypes of the embryos were reconstructed using an HMM based on the haplotype of the phased affected child as the reference (Additional file 1). In particular, the raw paternal/maternal haplotype of each embryo were compared with affected child, “i” for identical allele and “d” for different allele. The “i” and “d” can be randomly present within 500Kb region, which is not reasonable because the crossover is not recurrent within a relatively small region. Therefore, the HMM was used to reconstruct the refined haplotype of each embryo. Two states were assigned in each SNP loci in each embryo, “I” for refined identical allele and “D” for refined different allele. In this way, the discrete “i” and “d” were refined as continuous “I” or “D” block along the chromosome. Finally, the disease allele-linked haplotype block was then indicated, enabling the diagnosis of the disease allele carrier status of each embryo (Fig. 1, Additional file 1). The details of usage are provided in additional file (Additional file 2).

## Results

We applied our method in PGD in two families with genetic disorders to trace the inheritance of the disease variants from the mother and/or father to in vitro fertilization (IVF) embryos.

**Fig. 2:**
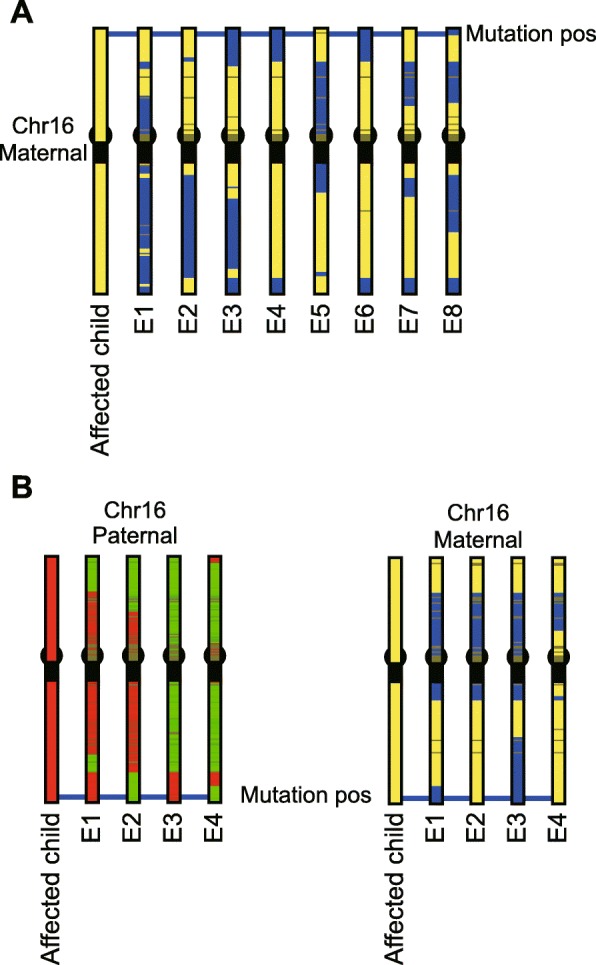
**a** Constructed maternal haplotype inheritance in an autosomal dominant disorder family in which the mother and child (daughter) were affected with polycystic kidney disease. The inherited paternal haplotype is not shown. The horizontal line indicates the mutation position in the *PKD1* gene on the chromosome. Yellow and blue represent maternal haplotype blocks that are in and not in the affected child’s genome, respectively. The E1, E2, E5, and E7 embryos had the mutation as determined by the inheritance of the same haplotype block as that identified in the affected child at the *PKD1* gene location. The E3, E4, and E6 embryos were free of the mutation because they inherited a different haplotype block than was found in the affected child at the *PKD1* gene location. A recombination was identified in the *PKD1* gene in E8. **b** Constructed haplotypes (paternal and maternal) of a family with an autosomal recessive disorder in which the father and mother were unaffected carriers, but a child (son) was affected with mucopolysaccharidosis. The horizontal line indicates the mutation position of the *GALNS* gene on the chromosome. Red and green represent paternal haplotype blocks in and not in affected child genome, respectively. Yellow and blue represent maternal haplotype blocks in and not in the affected child’s genome, respectively. The results show that embryos E1 and E3 had only the paternal mutations, and embryos E2 and E4 had only the maternal mutations. All embryos were therefore unaffected carriers

In the first family, the mother and daughter were affected with an autosomal dominant disorder, polycystic kidney disease. Genetic diagnosis of the mother and daughter showed an indel mutation (c.2473_2474delCG) in the *PKD1* gene. We applied our method to the eight embryos obtained from the parents. As shown in Fig. 2a, three (E3, E4, E6) of the eight embryos were free of the maternal mutation because these three embryos inherited a maternal haplotype block at the *PKD1* gene location that was different from the affected daughter. In this way, we could construct the inherited paternal and maternal haplotype blocks in each embryo and diagnose the mutation carrier status of each embryo. After diagnosis with haplotyping, E3 was transferred, resulting in the live birth of one unaffected baby in October of 2018.

In the second family, a son was diagnosed with an autosomal recessive disorder, type IVA mucopolysaccharidosis. Genetic diagnosis of the son showed two point mutations (paternal: c.374C > T, maternal: c.860C > G) in the *GALNS* gene that were inherited from his unaffected carrier parents. We applied our method to the four embryos obtained from the parents. The haplotyping results showed that E2 and E4 were free of the paternal mutation because these two embryos inherited a different paternal haplotype block at the *GALNS* gene location from the affected son. The E1 and E3 embryos showed a different maternal haplotype block at the *GALNS* gene location from the affected son and were, therefore, free of the maternal mutation (Fig. 2b). After diagnosis, embryo E1 was transferred, resulting in the live birth of one unaffected carrier baby in August 2019.

## Conclusion

We present the scHaplotyper, a convenient and accurate pipeline for haplotyping in preimplantation genetic diagnosis. By refining the haplotype with HMM, we can recalibrate the WGA artifacts in haplotyping, giving an accurate diagnosis of disease carrier status of embryos. We have applied this method to identify two families with genetic disorders, which resulted in two healthy babies free of the disease, indicating that this method is a reliable and accurate diagnostic test. The source code and related document are available at https://github.com/yzqheart/scHaplotyper

## Availability and requirements

**Project name:** scHaplotyper.

**Project home page:** https://github.com/yzqheart/scHaplotyper

**Operating system(s):** Linux.

**Programming language:** Perl, Python and Bash.

**Other requirements:** Perl SVG module, bcftools.

**License:** GPL v3.

**Any restrictions to use by non-academics:** None.

## Supplementary information

**Additional file 1.** The schematic diagram illustrating the HMM used in this study.

**Additional file 2.** The detailed description of the usage of the software.

## Data Availability

The software and related data are deposited in github repository: https://github.com/yzqheart/scHaplotyper
