# Supplementary figures and images for: scHaplotyper: haplotype construction and visualization for genetic diagnosis using single cell DNA sequencing data
Source: BMC Bioinformatics. 2020 Feb 1;21:41. doi: 10.1186/s12859-020-3381-5 (PMC6995221; doi:10.1186/s12859-020-3381-5)

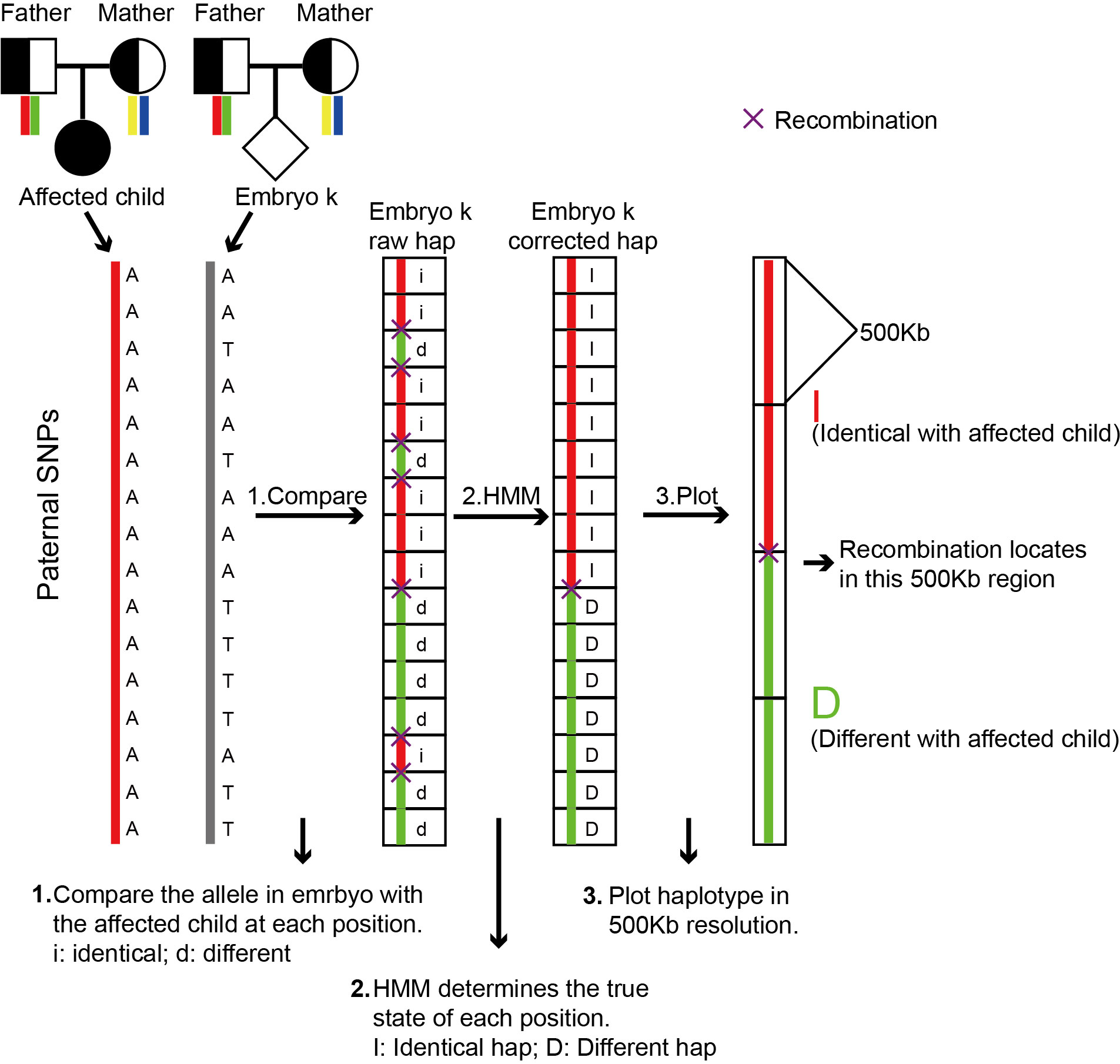

Supplement: Supplementary file 1 — Additional file 1. The schematic diagram illustrating the HMM used in this study. [file 12859_2020_3381_MOESM1_ESM.jpg]
