# Supplementary material for: scHaplotyper: haplotype construction and visualization for genetic diagnosis using single cell DNA sequencing data
Source: BMC Bioinformatics. 2020 Feb 1;21:41. doi: 10.1186/s12859-020-3381-5 (PMC6995221; doi:10.1186/s12859-020-3381-5)

Brief description

scHaplotyper is a method to perform haplotyping for genetic diagnosis using the single cell DNA sequencing data. First, trio father-mother-child and father-mother-embryo “core families” are constructed, and the affected child and embryos are phased to paternal and maternal inheritance. The affected child-inherited paternal and maternal haplotypes are shown as red and yellow, respectively. Second, the haplotypes of the embryos are reconstructed by regarding the affected child as a reference. The horizontal line indicates the mutation position on the chromosome. The embryo is diagnosed as carrying paternal and/or maternal mutations when the embryo inherits the same paternal and/or maternal haplotype block as the affected child does at the mutation position on the chromosome.

1. Input data

1.1 Sample information file

Sample information file includes four columns:
“Identity” indicates the identity of each sample.
“Sequenced” indicates whether the sample is sequenced.
“SampleName” indicates the sample name used in the analysis, this name must match the sample name in vcf file described below. “NA” represents the sample is not included in this analysis.
“CarryMutation” indicates whether the sample carry the known mutation. “NA” represents unknown.

Identity	Sequenced	SampleName	CarryMutation
Father	Yes	DKR-Father	Yes
Mother	Yes	HYJ-Mother	Yes
AffectedSon	Yes	DYZ-AffectedSon	Yes
AffectedDaughter	No	NA	NA
Embryo	Yes	E1	NA
Embryo	Yes	E2	NA
Embryo	Yes	E3	NA
Embryo	Yes	E4	NA

The first four lines include father, mother, affected son and affected daughter. The first two lines are required.

1.2 Mutation information file

The mutation information file includes three columns:
“MutationType” indicates the type of the disease. Four values are available: AutosomalDominant, AutosomalRecessive, XLinkedDominant, and

XLinkedRecessive.
"FatherMutPos" indicates the mutation position in father's genome. "NA" represents that father do not carry the mutation.
"MotherMutPos" indicates the mutation positon in mother's genome. "NA" represents that mother do not carry the mutation.

MutationType	FatherMutPos	MotherMutPos
AutosomalRecessive	chr16:88841040	chr16:88835251

1.3 Joint called vcf file

The joint called vcf file includes the genotype of each sample included in the analysis. In this study, we generated join called vcf file by using the GATK. The example is available at https://github.com/yzqheart/scHaplotyper/tree/master/example

2. Usage

./scHaplotyper SampleInfoFile MutationInfoFile VcfFile
The example is available at https://github.com/yzqheart/scHaplotyper

3. Output

The scHaplotyper produces a *results* directory. In the *results* directory, there are "chrN.pat.snp.het.filter.xxx.svg" for paternal mutation diagnosis and "chrN.mat.snp.het.filter.xxx.svg" for maternal mutation diagnosis. The first column represents the affected son/daughter, other columns represent the embryos. The red color represents the paternal mutation hap, the green color represents the paternal mutation-free hap (left panel). The yellow color represents the maternal mutation hap, and the blue color represents the maternal mutation-free hap (right panel). The horizontal line indicates the mutation position of this family.

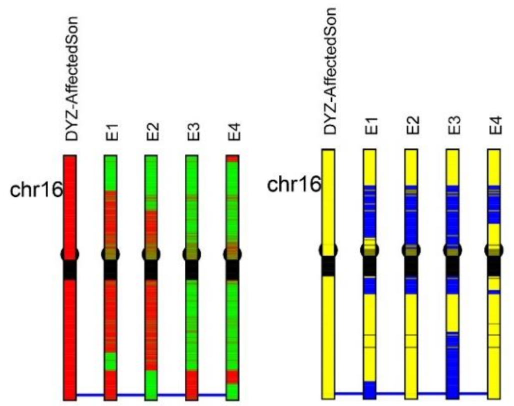

Supplement: Supplementary file 2 — Additional file 2. The detailed description of the usage of the software. [file 12859_2020_3381_MOESM2_ESM.pdf]
